# Prospection and Evaluation of (Hemi) Cellulolytic Enzymes Using Untreated and Pretreated Biomasses in Two Argentinean Native Termites

**DOI:** 10.1371/journal.pone.0136573

**Published:** 2015-08-27

**Authors:** Emiliano Ben Guerrero, Joel Arneodo, Raquel Bombarda Campanha, Patrícia Abrão de Oliveira, Mônica T. Veneziano Labate, Thaís Regiani Cataldi, Eleonora Campos, Angel Cataldi, Carlos A. Labate, Clenilson Martins Rodrigues, Paola Talia

**Affiliations:** 1 Instituto de Biotecnología, CICVyA, CNIA, INTA Castelar, Dr. N. Repetto y Los Reseros s/n, (1686) Hurlingham, Provincia de Buenos Aires, Argentina; 2 Consejo Nacional de Investigaciones Científicas y Técnicas (CONICET), Buenos Aires, Argentina; 3 Instituto de Microbiología y Zoología Agrícola, CICVyA, CNIA, INTA Castelar, Dr. N. Repetto y Los Reseros s/n, (1686) Hurlingham, Provincia de Buenos Aires, Argentina; 4 Embrapa Agroenergia, Parque Estação Biológica s/n, Av. w3 Norte (final), CEP 70770–901, Brasília, DF, Brazil; 5 Laboratório Multiusuários Centralizado de Genômica Funcional Aplicada à Agropecuária e Agroenergia, Escola Superior de Agricultura “Luiz de Queiroz”, Universidade de São Paulo, Av. Pádua Dias 11, CP 83, 13400–970, Piracicaba, SP, Brazil; University of Tennessee, UNITED STATES

## Abstract

*Saccharum officinarum* bagasse (common name: sugarcane bagasse) and *Pennisetum purpureum* (also known as Napier grass) are among the most promising feedstocks for bioethanol production in Argentina and Brazil. In this study, both biomasses were assessed before and after acid pretreatment and following hydrolysis with *Nasutitermes aquilinus* and *Cortaritermes fulviceps* termite gut digestome. The chemical composition analysis of the biomasses after diluted acid pretreatment showed that the hemicellulose fraction was partially removed. The (hemi) cellulolytic activities were evaluated in bacterial culture supernatants of termite gut homogenates grown in treated and untreated biomasses. In all cases, we detected significantly higher endoglucanase and xylanase activities using pretreated biomasses compared to untreated biomasses, carboxymethylcellulose and xylan. Several protein bands with (hemi) cellulolytic activity were detected in zymograms and two-dimensional gel electrophoresis. Some proteins of these bands or spots were identified as xylanolytic peptides by mass spectrometry. Finally, the diversity of cultured cellulolytic bacterial endosymbionts associated to both Argentinean native termite species was analyzed. This study describes, for the first time, bacterial endosymbionts and endogenous (hemi) cellulases of two Argentinean native termites as well as their potential application in degradation of lignocellulosic biomass for bioethanol production.

## Introduction

The interest in lignocellulosic ethanol has been increasing over the last years, mainly due to the global warming concerns and the uncertainty in the cost of petroleum. In this sense, the availability of environmentally friendly and sustainable biofuels is crucial.

Lignocellulose is the main component of the plant cell wall. This biomass produced by photosynthesis is the most promising and abundant renewable carbon source that can contribute to solve the current problems of energy. The absence of a low-cost technology aimed at obtaining fermentable sugars from lignocellulose hampers the use of plant biomass. The release of the sugars within cellulose fibers, which are embedded in the hetero-matrix of plant cell walls, generally requires pretreatment [1]. There are physical, chemical, biological and physicochemical pretreatments that enhance the hydrolysis of lignocellulose. Chemical treatments are among the most widely used. For instance, the acid pretreatment aims to solubilize the hemicellulosic fraction of the biomass and to make the cellulose more accessible to enzymes. For industrial applications, the diluted-acid pretreatment seems to be the most appropriate method; this chemical method has been used as a pretreatment for a wide range of lignocellulosic biomasses as well [2–4].

The lignocellulosic biomass is degraded by the synergistic action of several enzymes, such as endoglucanases, exoglucanases, β-glucosidases, xylanases, peroxidases and laccases [5]. Because of the recalcitrant nature of lignocellulose, the enzymatic degradation results in high costs of ethanol biofuel production [6]. Currently, the cost of (hemi) cellulases remains a key barrier to biofuel development. A wide range of naturally occurring lignocellulose-degrading enzymes that may help improve the biofuel industry is being thoroughly studied [7–10]. The use of recombinant enzyme technology may boost biofuel industry by developing more efficient enzymatic extracts and therefore reducing energy and cost inputs [11–13].

In Argentina and Brazil, the lignocellulosic biomass from sugarcane (*Saccharum officinarum* L.) bagasse exhibits high potential for biofuel production. Perennial grasses such as Napier grass (*Pennisetum purpureum* Schumach) are also selected for this purpose in both countries mainly because they can be sustainably grown and applied to local production during the interseason and because of their adaptability to marginal lands [14]. In this study, we have investigated the composition of both feedstocks and their saccharification potential for ethanol production.

Termites (Isoptera) are highly adapted for degrading cellulose, regarding their efficiency in view of their efficiency and the amount of lignocellulose consumed per year [15, 16]. This is due to the well-coordinated combination of its own mechanical and enzymatic machinery together with the gut endo-symbiotic cellulolytic microorganism (digestome). In addition, some higher termites also maintain exo-symbiotic associations (to their habitats) [15, 17–19]. A dual cellulolytic system is present in termites; in lower wood-feeding termites the cellulases are produced by the insect host and its gut flagellates, whereas in higher termites, host cellulases and hindgut bacteria are involved in wood digestion [15, 16, 18, 20].

The passage of food through the digestive tract takes about 24 h [15, 21]. The digestive process starts in the mandible, where the biomass is reduced to small particles sizes. This mechanical fragmentation of food is helped by enzymes of salivary glands. Then, the particles are moved through the intestinal tract, which consists of the foregut, midgut and hindgut, where several lignocellulolytic enzymes are secreted in each compartments [15]. In wood-feeding termite foregut the food is ground and the lignin is pretreated; then the food enters into the midgut, which is the main place for lignin-hemicellulose dissociation, esterase secretion and endogenous cellulose digestion [17, 20, 22]. Finally, it goes through the hindgut, which harbors symbiotic bacteria and archaea. This compartment is the main place for cellulose hydrolysis and many different hemicellulolytic enzymes within this organ are also produced by the bacterial symbionts.

The lignocellulolytic system in wood-feeding termites has some unique system advantages and can potentially serve as a model system to improve our current biomass bioconversion technology for fuels and chemicals.

The termites *Nasutitermes aquilinus* (Holmgren, 1910) and *Cortaritermes fulvicep*s (Silvestri, 1901) are widely distributed in the Parana River Basin in South America [23, 24]. The selection of these species was based on their relative abundance in the northeast Region of Argentina (NEA) and on their different nutritional habits. *N*. *aquilinus* constructs arboreal nests, feeds exclusively on wood and inhabits live and dead trees as well as decaying woods. On the other hand, *C*. *fulviceps* builds mounds and has a more varied diet, including roots, leaves and stems of gramineous plants, as well as wood [24, 25].

The aims of this work were to explore the diversity of culturable cellulolytic bacterial endosymbionts and the cellulolytic and hemicellulolytic activities in the two aforementioned Argentinean native termites. By focusing on these less studied Neotropical species, we attempted to discover new biological sources of enzymes with possible biofuel applications. Furthermore, the convenience of acid pretreatment and the suitability of regionally available biomasses for bioethanol production were investigated.

## Materials and Methods

### Biomass preparations

*Saccharum officinarum* L. bagasse (SOB) was kindly donated by The Jalles Machado mill (Goiás, Brazil) and *Pennisetum purpureum* var. Napier (PP) (Poales: Poaceae) was provided by the Brazilian Agricultural Research Corporation (Embrapa). The material was air dried and milled to an average particle size of 2 mm.

The biomasses were resuspended in diluted acid (0.5 N H_2_SO_4_) and pretreated in a 100°C water bath for 3 h. The solid fraction was separated from the liquid by vacuum filtration and washed twice with bidistilled water. The material used for the characterization was dried in an oven at 45°C for 8 h.

### Chemical composition

The acid insoluble lignin and carbohydrate content were determined following the protocols of the National Renewable Energy Laboratory (NREL), Golden, CO, USA.

Crude samples were sequentially extracted with water and ethanol, according to NREL methods [26] in order to quantify the extractives content.

The extractive-free and pretreated biomasses were subjected to a two-stage acid hydrolysis consisting of a first stage using 72 wt% sulfuric acid in a 30°C water bath for 1 h with frequent stirring, followed by a second stage with 4 wt% sulfuric acid in an autoclave for 1 h at 121°C. The resulting suspension was filtered and, subsequently, the filtrate was characterized by liquid chromatography coupled with refractive index detector. The content of glucan, xylan, arabinan and acetyl was established using liquid chromatography (Infinity 1290, Agilent, USA) equipped with a (G4204A, 1290 Quat Pump) quaternary solvent pump delivery, a (G1362A, RID) refractive index detector, a (G4226A, 1290 Sampler) sample injector with a column oven set at 55°C. The column used was a BioRad Aminex HPX-87H (300 x 7.8 mm, 9 μm) equipped with a Biorad Aminex Cation-H guard column (30 x 4.6 mm). The mobile phase was H_2_SO_4_ 5 mM used in isocratic mode. The flow rate was 0.6 mL/min and the total run time was 60 min. Agilent Open Lab with Data Analysis software (version A.01.01) was used for the operation of the LC system and for data processing. The soluble lignin in the filtrate was determined by ultraviolet (UV) absorption (240 nm and 40 L/g.cm), whereas the insoluble lignin was determined as the solid residue from hydrolysis subtracted out of its ash content.

### Scanning electron microscopy

Surface images of pretreated SOB and PP, with and without enzymatic digestion, were obtained by scanning electron microscopy (SEM) and compared with the untreated material. The samples were directly placed on graphite layer and observed at magnifications of x800–2000 in a scanning electron microscope model FEI Quanta-250 (FEI Co., Netherlands). Several images were obtained from different areas of the samples (at least 20 images per sample) to confirm the reproducibility of the results. Microscopy observations were made at the Microscopy Laboratory of CICVyA, INTA, Bs As, Argentina.

### Insect collection

The Argentinean termite species *Nasutitermes aquilinus* and *Cortaritermes fulviceps* belong to Order Isoptera, Family Termitidae and Subfamily Nasutitermitinae. Both species are widely distributed in the northeast region of Argentina (NEA). *N*. *aquilinus* were collected from *Enterobolium contortisiliquun* live trees of the province of Corrientes, Argentina (S 27° 28’ 30”: W 58° 46’ 59.43”). *N*. *aquilinus* has also been found in *Eucalyptus grandis* afforestation and was reported attacking other eight tree species from this province: *Eucalyptus camaldulensis*, *E*. *tereticornis*, *Grevillea robusta*, *Melia azedarach*, *Peltophorum dubium*, *Schinopsis balansae*, *Sorocea sprucei* and *Tabebuia heptaphylla* [25]. This species feeds on woods and inhabits on live and dead trees and within decaying woods.

*C*. *fulviceps* were collected from the province of Corrientes, Argentina (S 27° 26’ 58.26”: W 58° 44’ 17.64”) inside a mound located in *Elionurus muticus* grassland; these communities have been partially affected by cattle grazing. This species has also been reported attacking *Acrocomia totai* palm stems and *Eucalyptus grandis* live trees, but these situations seemed to be rare [24, 25].

Worker caste specimens were field-collected from the province of Corrientes, and stored at -20°C until processing. The insects were surface sterilized with 70% ethanol and their whole guts were dissected under binocular microscope. The dissected whole guts were ground in bidistilled water or 5 mM Tris-HCl pH 7.6, homogenized by vortexing and centrifuged at 12,000 *g* for 10 min at 4°C. A protease inhibitor cocktail (Thermo Scientific, USA) (1μl/mL) was added to the supernatant, hereafter referred as gut extract (GE), until use.

The termites were collected with the permission of the Direction of Natural Resources of Ministry of Tourism of the province of Corrientes (permission number 845/13). No endangered or protected species were used in this study.

### Culture conditions

The homogenates containing insect endosymbiotic bacteria were grown on liquid minimal medium (MM) according to Hanking and Anagostakis [27], supplemented with different cellulosic substrates: 1% (w/v) carboxymethylcellulose (CMC) low viscosity (P.M: 90000), 0.5% (w/v) xylan, 0.5% SOB (untreated and pretreated) and 0.5% PP (untreated and pretreated) as the sole carbon source. CMC was chosen based on its suitability for detection of endoglucanase activity. The cultures were grown at 37°C in aeration for a week.

### Qualitative screening of cellulase and hemicellulase activities

A qualitative assay of degradation on solid medium was performed to evaluate the endoglucanase and xylanase activities. Aliquots of each culture supernatant (10 μL) were seeded on the surface of the plates. The plates containing MM—1% CMC and MM—1% xylan from beechwood (Sigma, USA) were incubated at 37°C for 16 h. The endoglucanase and xylanase activities were assayed by checking their ability to form degradation halos detected by Congo red staining.

### Enzyme assays and protein determination

The enzymatic activity and protein concentration were assessed in culture supernatants of gut endosymbionts of both termite species grown on the previously described cellulosic substrates. Ten milliliters of each culture were centrifuged at 12,000 *g* at 4°C for 20 min. The cultured supernatants were filtered through 0.2 μm filter (Ministart, Sartorius Stedim Biotech, USA) and used to assess endoglucanase and xylanase activities. The release of reducing sugar was measured by the 3,4-dinitrosalycylic acid method adapted to small reaction volumes [28] from hydrolysis of the following substrates: CMC, xylan, SOB and PP. Hydrolysis reactions were carried out by triplicate. The assays were performed using 100 μL of culture supernatant and 100 μL of 1% CMC or 1% xylan, in 0.1 M phosphate citrate buffer pH 5 and incubated at 50°C for 60 min (CMC) or 30 min (xylan). A culture supernatant without substrate and the substrate in buffer (without enzymes) were analyzed as negative controls, whereas a commercial cellulase from *Aspergillus niger* (Sigma, USA) was used as a positive control. The absorbance readings were compared to glucose or xylose standard curves ranging from 0.05 to 2.5 mg/mL. The enzyme activity (U/mL) was determined considering 1 IU equivalent to 1 μmol of glucose or xylose released per min under the assayed conditions.

To calculate the specific activity, we measure the protein concentration by the bicinchoninic acid assay (BCA) (Thermo Scientific, USA), with bovine serum albumin (BSA) as the standard. The statistical significance was determined by the Two-Way Analysis of Variance using GraphPad Software (San Diego, CA, USA).

### Detection of cellulases and hemicellulases by zymography

To detect proteins with endoglucanase and xylanase activities, we performed SDS-PAGE zymograms in whole gut extracts (GE) and in supernatants from bacterial endosymbionts (SN) grown in 1% CMC, 0.5% SOB and 0.5% PP, concentrated using Corning Spin-X UF Concentrators (Corning, USA). The samples were loaded into 10% SDS-polyacrilamide gels containing 0.5% CMC or 0.5% xylan. After electrophoresis, the gels were treated as previously described [29]. Briefly, they were washed two times with 0.04 M Tris-HCl, pH 7.6 for 1 h each and incubated at 4°C over night. Then, the gels were washed again and incubated at 37°C for 2 h. Finally, they were stained with Congo red for 15 min and subsequently destained in 1M NaCl.

### Two-dimensional (2D) gel electrophoresis

A two-dimensional (2D) gel electrophoresis from SN–CMC–cultures from *N*. *aquilinus* was performed according to the following protocol:

Proteins present in the supernatant of a 7-day old culture were concentrated and desalted by precipitation with 10% trichloroacetic acid (TCA). The protein pellets were resuspended with a rehydration buffer (8 M urea, 2% CHAPS, 0.5% IPG buffer pH 4–7 and 20 mM DTT). A volume of 150 μL containing 400 mg protein was used to rehydrate a 7 cm immobilized linear gradient strip (pH 4–7) (Immobiline DryStrips, GE Healthcare) for 6 h at room temperature. Isoelectric focusing was carried out with an Ettan IPGphor 3 system (GE Healthcare), by using the following programme: 1, 0.2 kV h at 300 V; 2, 0.3 kV h (gradient) at 1000 V; 3, 4.5 kV h (gradient) at 5000 V; 4, 30 kV h at 5000 V.

After the isoelectric focusing was complete, the strip was equilibrated for 10 min in equilibrium buffer (2% SDS, 50 mM Tris/HCl, pH 8.8, 6 M urea, 30% glycerol, 0.002% bromophenol blue and 1% DTT). The strip was then overlaid onto 10% SDS-PAGE and, after the electrophoresis was complete, the gel was stained with colloidal Coomassie blue.

Ten of the most intensively stained spots were cut from the 2D-gel electrophoresis and identified by mass spectrometry sequencing.

### DNA extraction and PCR amplification

Total DNA from bacterial endosymbionts grown in MM-CMC was extracted by the CTAB method developed by Doyle & Doyle [30].

The bacterial near-full-length 16S rRNA gene sequences (approximately 1400 bases) were amplified with primers fD1 (CCGAATTCGTCGACAACAGAGTTTGATCCTGGC TCAG) and rD1 (CCCGGGATCCAAGCTTAAGGAGGTGATCCAGCC), as specified by Weisburg et al. [31]. PCR was carried out in 25 μl (final volume) with a MiCycler Thermal Cycler (BioRad, USA). Each reaction comprised 50 ng of total DNA in 1X PCR buffer including 1.5 mM MgCl, 0.2 mM dNTPs, 0.25 mM of each primer, 0.75 U of Taq DNA polymerase (Invitrogen, USA). The PCR conditions were, an initial denaturation step (10 min at 95°C), followed by 30 cycles of amplification (20 s at 95°C, 20 s at 45°C and 30 s at 72°C), and a terminal extension step (3 min at 72°C).

The amplified DNA products were resolved by agarose gel electrophoresis (1%), stained with ethidium bromide and photographed under UV light (302 nm). The PCR amplicons were purified with QIAquick purification kit (Qiagen, USA).

### Cloning and sequencing of the 16S rRNA gene

The purified amplification products were cloned into *Escherichia coli* DH5-α-competent cells using the TA cloning pGEM-T Easy vector (Promega, USA) according to the manufacturer’s instructions. The plasmid DNA from these cultures was isolated with the QIAprep Spin Miniprep Kit (Qiagen, USA).

Sequencing was performed by the Biotechnology Institute Sequencing Service, INTA Castelar with an ABI 3130xl Capillary DNA sequencer (Applied Biosystems, USA). The clones were sequenced using specific fD1 and rD1 primers. All nucleotide sequences were checked for sequence quality and putative chimeras with DECIPHER's Find Chimeras (http://decipher.cee.wisc.edu/FindChimeras.html) [32]. The partial sequences were compared by the specific ribosomal DNA database Ribosomal Database Project II (RDP) (http://rdp.cme.msu.edu/) and GenBank using BLAST (http://www.ncbi.nlm.nih.gov/) program.

### Estimation of bacterial diversity

The species richness was defined as the number of OTUs present in each sample. The rarefaction curves at 97%, 95%, 90% and 80%, Chao1 estimator, abundance-based coverage estimator (ACE) [33] and the α-diversity estimators (the Shannon and Simpson indices) were calculated with the Mothur program [34].

The β-diversity (partitioning of diversity among communities) was estimated based on the number of shared species by LIBSHUFF algorithm with the Mothur program [34].

The diversity and richness indices were estimated based on 3% differences in nucleic acid sequence alignment with 97% confidence intervals as calculated by the Mothur program [34].

### Phylogenetic analyses

The 16S rRNA sequences were assigned to taxonomic groups based on the annotations available at Ribosome Database Project (RDP) and NCBI, and aligned with the MEGA program version 5 [35]. Reference sequences for each genus, retrieved from GenBank, were added.

Maximum likelihood (ML) trees were built assuming a Tamura-Nei, gamma distributed model. Branch support was calculated by bootstrapping, performing 1000 resampling iterations.

### Enzyme sequencing and identification

Cellulase bands were separated by SDS-PAGE gels (10%) containing 1% CMC and then stained with Congo red or Coomasie blue. The bands corresponding to active cellulases were excised from the gels, cut in small pieces, destained, reduced and alkylated, according to Schevchenko et al. [36]. Subsequently, these bands were subjected to tryptic digestion with 10 ng/mL of Trypsin Gold (Promega Corporation, Wisconsin, USA), according to Celedon et al. [37]. The digestion reaction was interrupted by incubating the gel spots into the corresponding buffer [50 μL of 50% (v/v) acetonitrile (ACN), 5% (v/v) formic acid (FA)]. The peptides were then extracted twice with [50 μL of 60% (v/v) methanol, 1% (v/v) FA], twice with [50 μL of 50% (v/v) ACN and MS-grade-water, 1% (v/v) FA] and once with [50 μL of ACN (100%), 1% (v/v) FA]. All supernatants were withdrawn, combined and vacuum dried. The peptides were desalted with the reversed-phase C18ZipTip column from Millipore, according to the manufacturer’s instructions. The purified peptides were eluted in 50% (v/v) ACN and MS-grade-water, 0.1% (v/v) trifluoracetic acid (TFA), mixed with a solution of 5 mg/mL of α-ciano-4-hydroxycinnamic acid (CHCA) matrix prepared in [50% (v/v) ACN and MS-grade-water, 0.1% (v/v) trifluoracetic acid (TFA). Subsequently, the proteins were loaded onto each spot of the sample plate and the plate was left to dry at room temperature for 30 min.

The mass spectrometry analysis was performed in the positive ion reflector mode by accumulating data from 1000 laser shots (200/spectrum), using 5200 Hz (MS) and 6000 Hz (MS/MS). Peptide masses were measured based on their *m/z* between 900–4000 with focus in 2000, using the Matrix Assisted Laser Desorption/Ionization System (MALDI-TOF/TOF 5800 from ABSciex).

The instrument was calibrated using Calibration Mixture 1 (Mass Standards Kit for Calibration-ABSciex TOF/TOF). The operating conditions of the detector were: detector voltage multiplier = 0.7, final detector voltage = 2100, pulse rate (Hz) = 400. The control of the instrument, data acquisition in the automatic mode, and data evaluation were performed wih the TOF/TOF Series Explorer Software v. 4.1.0.

The resulting spectra were processed with the ProteinPilot Software (ABSciex) combined with MASCOT MS/MS Ion Search (www.matrixscience.com) and the sequences were compared with those in the NCBI, and UniProt databanks.

### Statistical Analysis

Data were expressed as the mean ± standard deviation of three replicates. Enzymatic activity data were analyzed for statistical significance by Two-Way Analysis of Variance (ANOVA) followed by Tukey´s multiple comparison post test using GraphPad Prism 6 for Windows, GraphPad Software (San Diego, CA, USA).

The chemical composition data are presented as weight percent on dry as the mean ± standard deviation of two replicates. The differences between data were statistically analyzed using Student t-test. STATISTICA software (StatSoft, Tulsa, OK) was used. The significant differences were tested considering *p* value < 0.05.

### Nucleotide sequence accession numbers

Partial 16S rRNA nucleotide sequences determined in this study were deposited at GenBank under accession numbers [GenBank: KJ933510- KJ933529].

## Results and Discussion

### Untreated and pretreated biomass composition

The carbohydrate content of biomasses is an essential factor in the conversion of sugars into ethanol. To analyze the suitability of two common feedstocks in Argentina and Brazil, we selected and assessed the biomass carbohydrate composition of *Saccharum officinarum* bagasse (SOB) (sugarcane bagasse) and *P*. *purpureum* (PP) (Napier grass) (Table 1). For the comparison of xylan, glucan, arabinan, acetyl and lignin content in both assessed biomasses, we used a NREL methodology [26]. Hemicellulose fractions were analyzed after pretreatment to evaluate the changes in monosaccharide composition (Table 1).

**Table 1:** Percentage of biomass composition of the untreated or pretreated (-P) *Saccharum officinarum* bagasse (SOB) and *Pennisetum purpureum* (PP)*.

Sample	Ashes	Structural proteins	Total extractives	Total Lignin	Glucan	Xylan	Arabinan	Acetyl	Total
**SOB**	0.62 (± 0.45)^a^	0.20 (± 0.04)^a^	6.09 (± 0.28)	26.49 (± 0.34)^a^	35.50 (± 3.28)^a^	15.27 (± 1.00)^a^	1.56 (± 0.05)^a^	5.64 (± 0.31)^a^	91.37
**SOB-P**	0.74 (± 0.00)^a^	0.27 (± 0.16)^a^	-	32.66 (± 1.03)^b^	48.97 (± 1.21)^b^	8.74 (± 0.22)^b^	0.73 (± 0.02)^b^	2.15 (± 0.03)^b^	94.25
**PP**	1.95 (± 0.15)^A^	0.42 (± 0.02)^A^	11.70 (± 0.06)	25.19 (± 0.06)^A^	33.87 (± 1.31)^A^	11.98 (± 0.41)^A^	1.57 (± 0.03)^A^	4.01 (± 0.11)^A^	90.69
**PP-P**	2.21 (± 0.02)^A^	0.53 (± 0.01)^B^	-	31.66 (± 0.91)^B^	49.61 (± 1.31)^B^	8.17 (± 0.28)^B^	0.65 (± 0.03)^B^	2.10 (± 0.07)^B^	94.92

*Values with the same lower case in the same column are not significantly different at *p* < 0.05; values with the same capital letter in the same column are not significantly different at *p* < 0.05.

The hemicellulose fraction showed the same composition in both untreated biomasses, including the main constituents: xylan, arabinan and acetyl derivatives. However, SOB presents higher content of xylan (Table 1). On the other hand, pretreated biomasses (SOB-P and PP-P) showed a reduction in the hemicellulose fraction and an increase in the lignin content. Furthermore, the enrichment in cellulose (glucan) also was a direct consequence of the removal of hemicellulose (Table 1). This finding indicates that the diluted acid pretreatment removed part of the hemicellulose fraction of *S*. *officinarum* bagasse and *P*. *purpureum*. Additionally, the Student’s t-test indicated that the content of main compounds of the pretreated and non-pretreated biomasses was significantly different at 95% confidence level (Table 1). These results are in agreement with those reported by Lima and coauthors [14], who used acid, alkaline, sulfite and hot water pretreatment in sugarcane bagasse and several grasses including *P*. *purpureum*. In their study, they demonstrated that sulfuric acid was the most effective compound for hemicellulose removal, whereas sodium hydroxide was more suitable for removing hemicellulose and lignin content in *P*. *purpureum*.

The need of developing a more efficient and cheaper pretreatment to improve the enzymatic saccharification of cellulose is a consensus among researchers [14, 38, 39]. However, lignocellulosic biomasses are complex and heterogeneous among different species and consequently different pretreatments should be optimized for each feedstock.

### Morphological changes produced by pretreatment

To improve enzymatic digestibility, we evaluated the effect of diluted acid pretreatments on the structure of the two selected biomasses by scanning electron microscopy.

**Fig 1:**
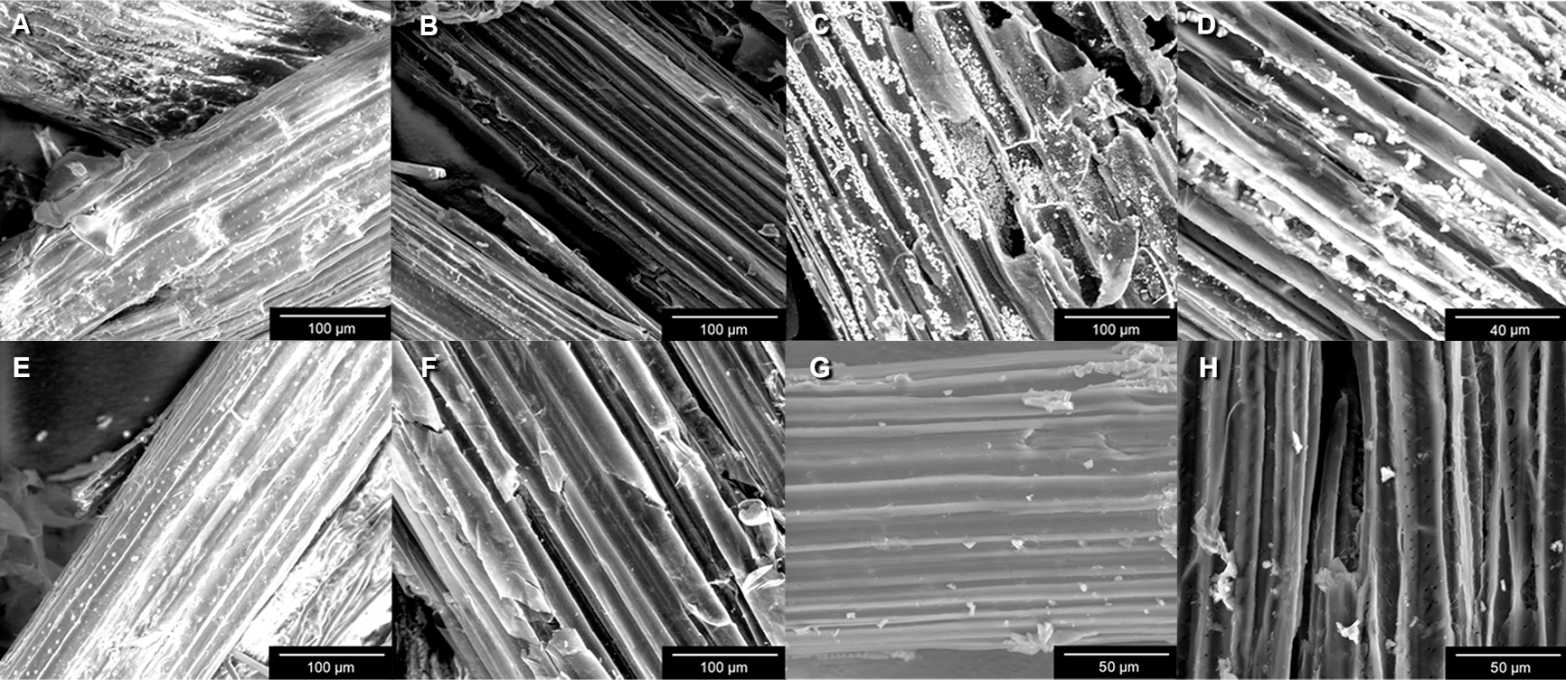
SEM photographs. (A) Untreated sugarcane bagasse (800X). (B) Pretreated sugarcane bagasse (800X). (C) Pretreated *S*. *officinarum* bagasse—*N*. *aquilinus* (800X). (**D)** Pretreated *S*. *officinarum* bagasse—*C*. *fulviceps* (2000X). (E) Untreated *P*. *purpureum* (800X). (F) Pretreated *P*. *purpureum* (800X). (**G)** Pretreated *P*. *purpureum*—*N*. *aquilinus* (1500X). (H) Pretreated *P*. *purpureum*—*C*. *fulviceps* (1600X).

The surface of untreated *S*. *officinarum* bagasse showed a continuous covering layer, probably composed by hemicellulose and lignin (Fig 1A). By contrast, the pretreated material displayed less cohesion between fibers and cellulose bundles became more evident (Fig 1B). This could be due to the type of pretreatment used, because the diluted acid pretreatment removed high levels of hemicellulose as described above. After the action of hydrolytic enzymes (both endogenous and of microbial origin) present in the gut from both termite species, we observed further breaking and separation of the fibers of this biomass (Fig 1C and 1D).

In the case of *P*. *purpureum* the effects of the pretreatments were weaker; the bundles were evident only in some regions of the sample surface (Fig 1E and 1F). After exposure to hydrolytic enzymes, *P*. *purpureum* samples also appeared to be relatively less degraded in comparison to those of SOB (Fig 1G and 1H). This result suggests that for this feedstock a more severe pretreatment is needed to improve the separation of the cellulose fibers in order to allow further access to enzymes. However, it is important to monitor possible losses in cellulose content in this optimization step.

### Enzymatic Activities

The (hemi) cellulolytic activities (endoglucanase and xylanase) were assessed in gut extracts (GE) and cell-free supernatants (SN) from cultures grown on carboxymethylcellulose (CMC), xylan, *S*. *officinarum* bagasse (SOB) and *P*. *purpureum* (PP) for both termite species. Bacterial growth was observed in every assayed condition, which suggests the presence of microorganisms expressing the enzymes needed to breakdown cellulosic substrates.

**Fig 2:**
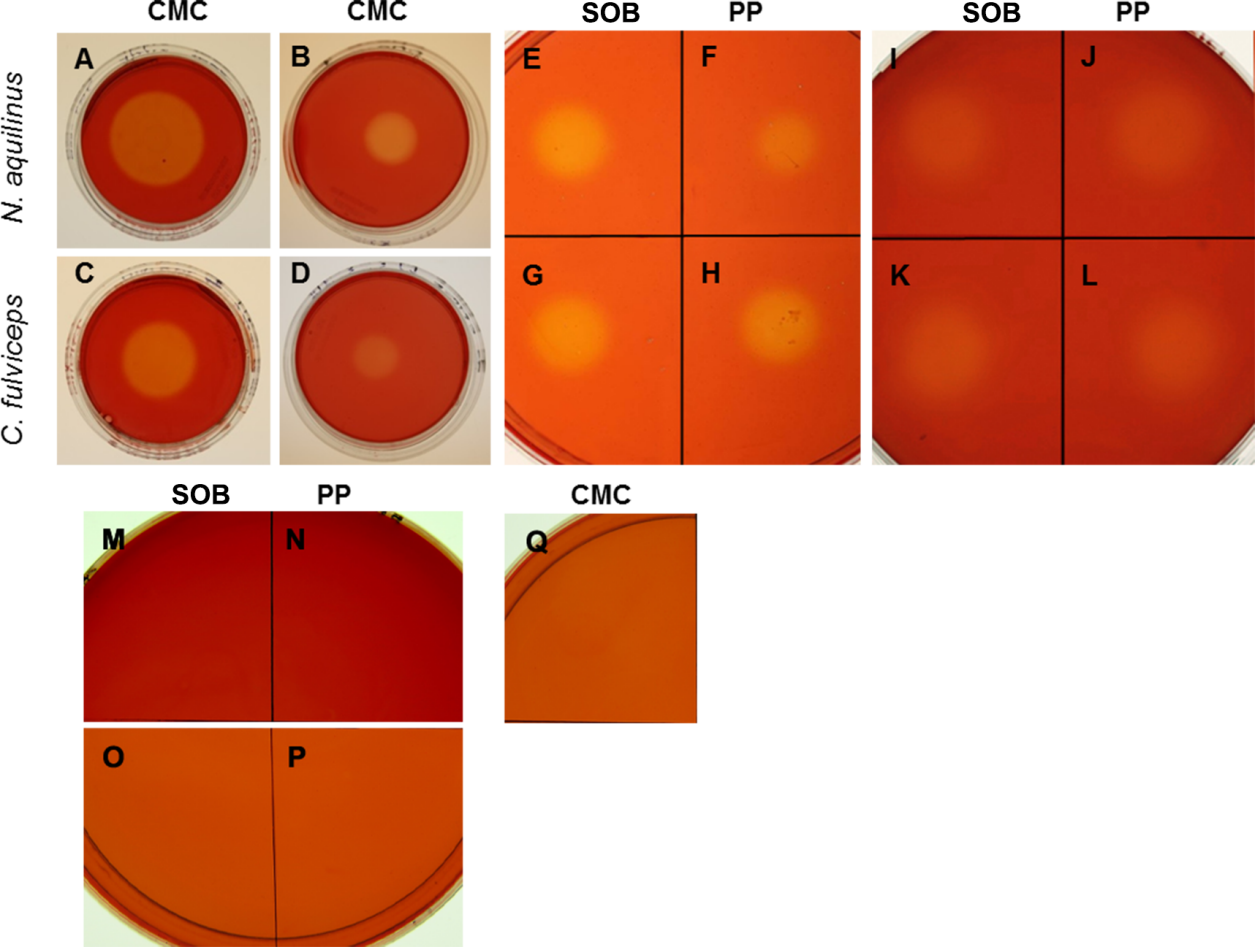
Qualitative endoglucanase and xylanase activity assay. (A) GE—*N aquilinus*. (B) SN—CMC—cultures from *N*. *aquilinus*. (C) GE—*C*. *fulviceps*. (D) SN—CMC—cultures from *C*. *Fulviceps*. (E) SN—SOB—cultures from *N*. *aquilinus*. (F) SN—PP—cultures from *N*. *aquilinus*. (**G)** SN—SOB—cultures from *C*. *fulviceps*. (H) SN—PP—cultures from *C*. *fulviceps*. (I) SN—SOB—cultures from *N*. *aquilinus*. (J) SN—PP—cultures from *N*. *aquilinus*. (K) SN–SOB—cultures from *C*. *fulviceps*. (L) SN-PP—*C*. *fulviceps*. (M) Negative control SOB medium in CMC plate. (N) Negative control PP medium in CMC plate. (O) Negative control SOB medium in Xyl plate. (P) Negative control PP medium in Xyl plate. (Q) Negative control CMC medium in CMC plate.

The xylanase and endoglucanase activities were evidenced in all treatments by a zone of clearance around the sample drop, which indicated hydrolytic degradation (Fig 2). Regarding CMC hydrolysis, GE and SN-CMC from *N*. *aquilinus* showed a clear zone diameter, which was greater than those from *C*. *fulviceps* (Fig 2A–2D). Using SN from cultures grown on SOB (SN-SOB), we detected a similar degradation halo for both termites. By contrast, SN-PP from *N*. *aquilinus* exhibited a greater degradation halo than SN-PP from *C*. *fulviceps* (Fig 2E–2H). The xylanase activity of both SN-PP and SN-SOB showed similar degradation halos regardless of the termite species (Fig 2I–2L).

**Fig 3:**
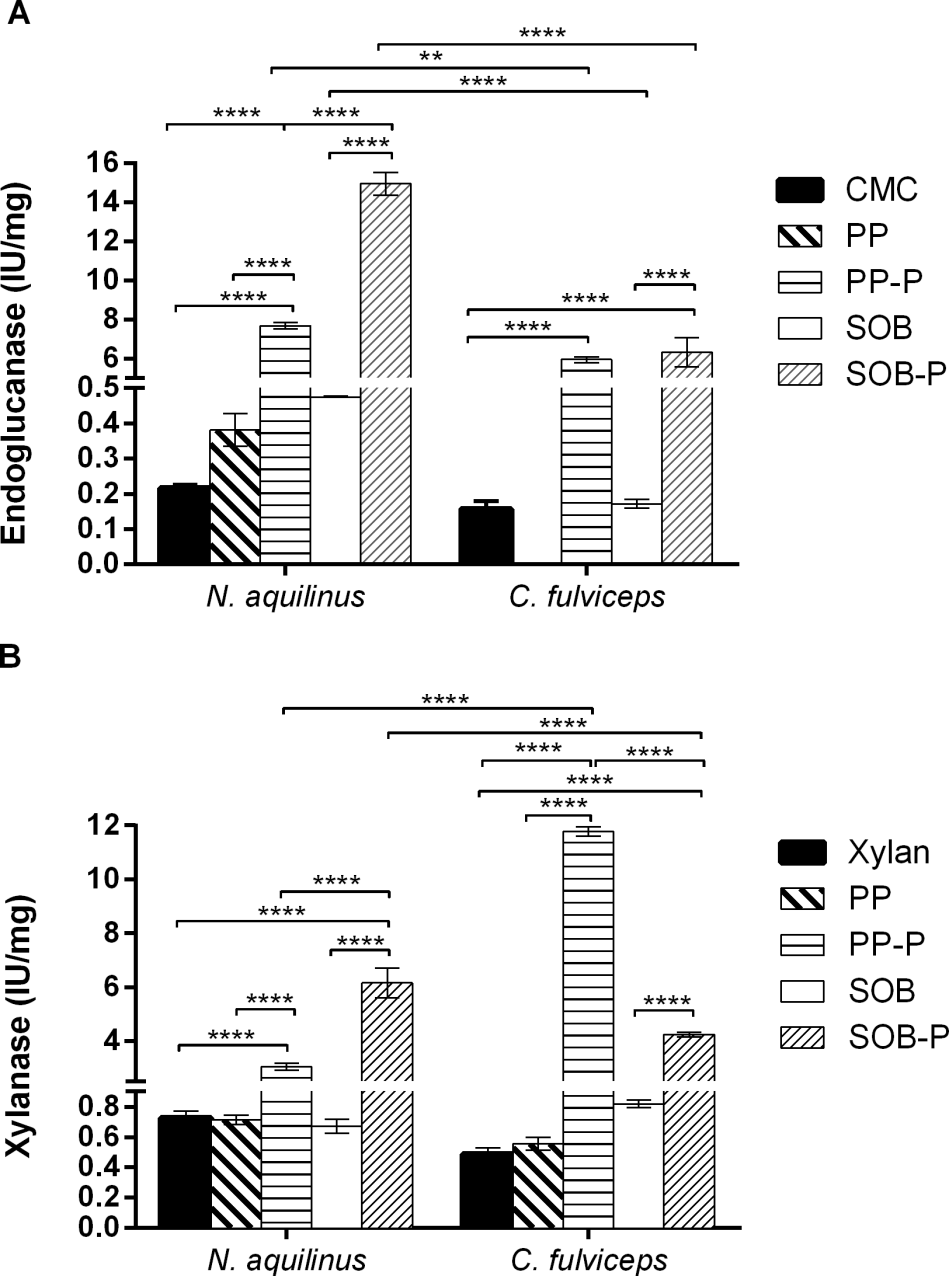
Quantitative enzymatic activities in cell-free culture supernatants. Endoglucanase. (B) Xylanase. Data were calculated as the means ± SD of triplicates assay ****P<0.0001; **P<0.01.

The endoglucanase and xylanase activities in SN from cultures grown on CMC and xylan untreated and pretreated biomass (SOB and PP) were measured quantitatively using the dinitrosalicylic acid (DNS) method (Fig 3). We applied Two-Way Analysis of Variance (ANOVA) to analyze the endoglucanase and xylanase activities for the five different supernatant conditions in the two termite species. The results showed significant differences (*P<*0.0001) of endoglucanse and xylanse activities (Fig 3).

The cultures from *N*. *aquilinus* pretreated biomass (SOB-P) showed the highest endoglucanase activity when all treatments were compared. The cultures from *C*. *fulviceps* showed the highest endoglucanase activities with both pretreated biomasses (PP-P and SOB-P), with no significant differences between them (Fig 3A). No endoglucanase activity was detected for *C*. *fulviceps* with untreated PP biomass.

Regarding the xylanase activities, the cultures from *N*. *aquilinus* showed significantly higher xylanase activity for pretreated SOB (SOB-P) relative to other treatments, whereas *C*. *fulviceps* exhibited the highest xylanase activities in PP-P (Fig 3B). As expected, the use of pretreated biomass significantly improved the enzymatic activity in all tested conditions.

With respect to the assessed termites, the cultures from *N*. *aquilinus* had the highest endoglucanase activity when grown on pretreated SOB (SOB-P), whereas those from *C*. *fulviceps* showed the highest xylanase activity when grown on pretreated PP (PP-P). These results strongly suggest that termites with non-wood feeding habits such as *C*. *fulviceps* can also constitute an interesting source of enzymes, specifically hemicellulases, for the digestion of lignocellulosic materials (Fig 3).

The detection of enzymatic activity is directly dependent on the sample preparation, the medium and the concentration of the specific substrates. In this sense, although the activity values obtained in this work may be low, they are in concordance with those reported in other studies [29, 40–43]. Searching for novel enzymes in Brazilian termites, Lucena et al. [44] reported similar hydrolytic capacity on micronized SOB and other substrates (approximately 0.2 IU/mg), as those described here, for commercial substrates and untreated biomass. Nevertheless, it is interesting to note that an increase of an order of magnitude was observed when the pretreated biomasses were used as substrates for culture growth.

### Detection of cellulases and xylanases by zymography and two- dimensional gels electrophoresis (2D-gel)

**Fig 4:**
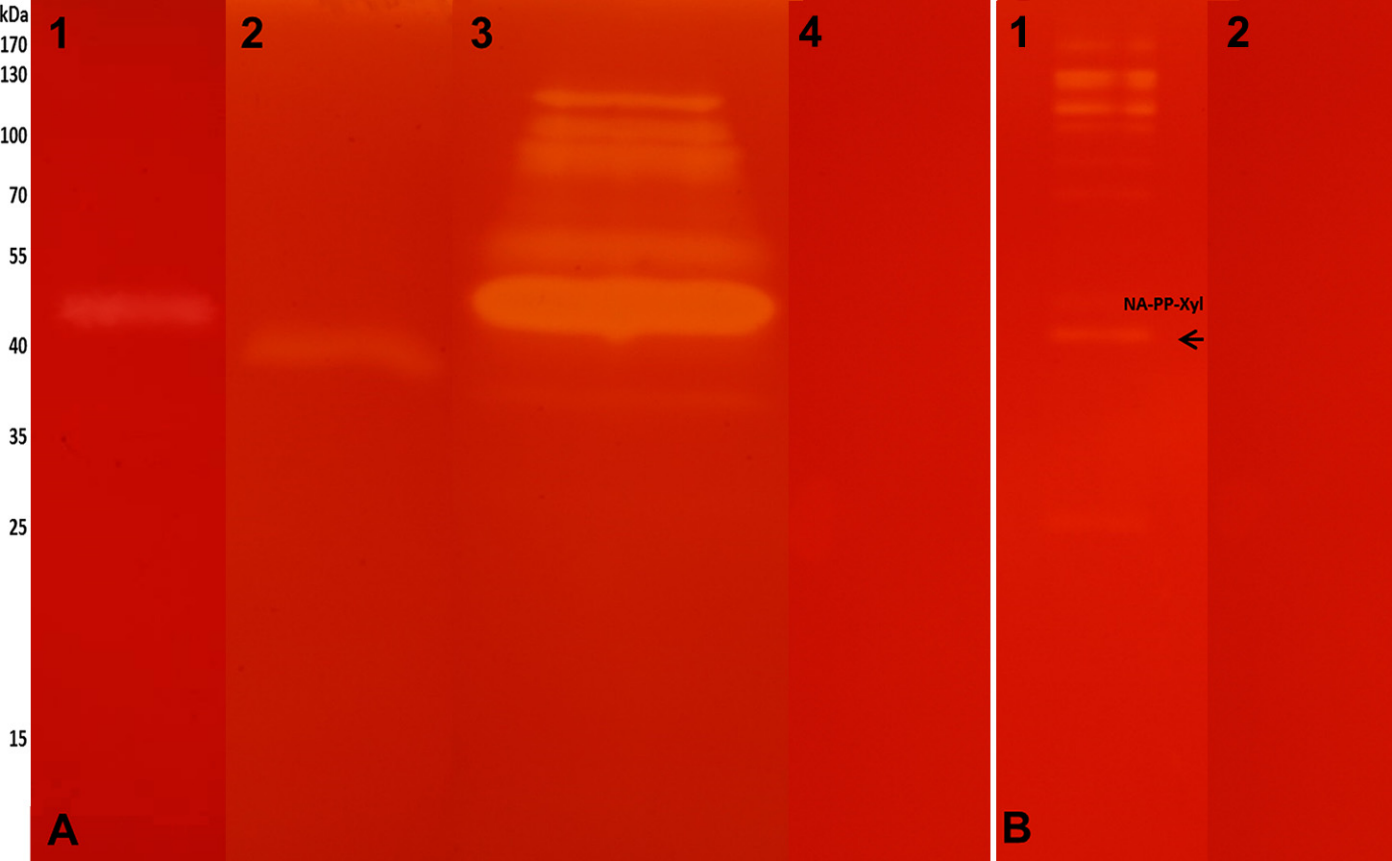
Zymogram. (A) **SDS-PAGE-CMC** lane 1 GE–*C*. *fulviceps*, lane 2 GE—*N*. *aquilinus*, lane 3 SN—cultures from *N*. *aquilinus*, lane 4 negative control, medium CMC. (B) **SDS-PAGE-xylan** line 1 SN—cultures from *N*. *aquilinus P*. *purpureum*, lane 2 negative control, medium PP. The arrows show the bands analyzed by de novo sequencing peptide identification.

**Fig 5:**
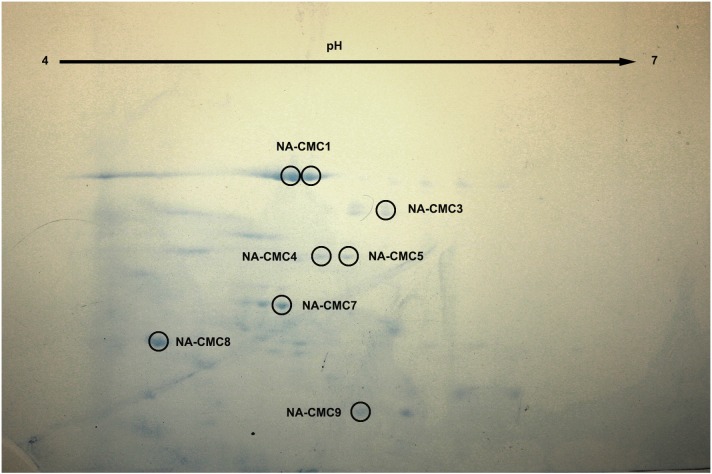
Coomassie blue-stained 2D gel image of protein spots from SN—cultures from *N*. *aquilinus*. The gel spot names, relative to the proteins identified by mass spectrometry are indicated with a circle and their protein accession number are shown in Table 3.

To further identify cellulases and hemicellulases in *N*. *aquilinus* and *C*. *fulviceps*, we analyzed GE and SN from both insects. The zymograms of GE-CMC consistently showed the presence of at least one endoglucanase in each termite species, with molecular weights of 45 kDa and 40 kDa (*N*. *aquilinus* and *C*. *fulviceps*, respectively) (Fig 4A, lane 1 and 2). In the SN-CMC from cultures from *N*. *aquilinus*, six relevant protein bands (around 45–130 kDa) with cellulolytic activity were observed (Fig 4A, lane 3). When xylan was used as substrate for zymography, several distinct xylanolytic protein bands were detected in SN from cultures of *N*. *aquilinus* endosymbionts grown on untreated PP (Fig 4B). However, no activity was observed in the SN from cultures from *C*. *fulviceps* bacterial cultures grown on CMC, xylan and untreated SOB. The detected molecular weights are comparable to those reported for endoglucanases and xylanases from insects [45–47]. Separation of these proteins by 2D-gel electrophoresis in SN-CMC cultures from *N*. *aquilinus* has allowed the identification of about eight relevant proteins stained with Coomassie blue (Fig 5).

### Bacterial diversity and phylogenetic analysis

A genomic sequence analysis of the 16S ribosomal RNA gene was performed to study bacterial diversity of two cellulolytic enrichment cultures from *N*. *aquilinus* and *C*. *fulviceps* guts, using CMC as carbon source.

**Fig 6:**
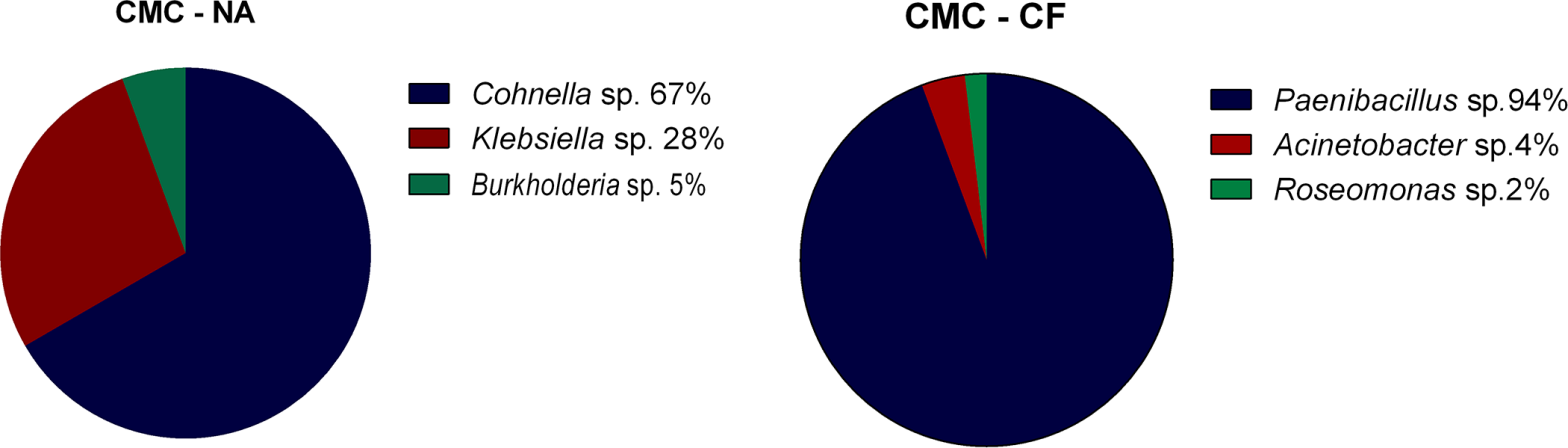
Bacterial diversity assessed by 16S rRNA cultured clone library. (A) CMC—cultures from *N*. *aquilinus*. (B) CMC—cultures from *C*. *fulviceps*.

The bacterial 16S rRNA gene was successfully amplified, cloned and sequenced from cultured termite gut. The classification of bacterial diversity represented in the clone libraries is depicted in Fig 6.

The microorganisms identified by the 16S rRNA approach belong to the phyla *Firmicutes* and *Proteobacteria* (alpha, delta and gamma-proteobacteria). Fig 6 shows the relative abundance of the taxa identified in the different samples. The predominant bacterial genus in cultures from *N*. *aquilinus* was *Cohnella* (67% of the sequenced clones), followed by *Klebsiella* (28%) and *Burkholderia* (5%). The predominant genus in cultures from *C*. *fulviceps* was *Paenibacillus* (94%), whereas the remaining 6% consisted of *Acinetobacter* (4%) and *Roseomonas* (2%). According to previous findings, most of these genera have been reported as cellulolytic in a variety of environments. Since novel undescribed species are likely to be found within these genera, the bacteria associated to *N*. *aquilinus* and *C*. *fulviceps* are worthy of further characterization. Moreover, these bacterial species are probably specifically adapted to the termite gut and might be involved in complex symbiotic relationships with other gut microorganisms [48–50].

The cellulolytic and xylanolytic activity among *Paenibacillus* spp. is well known [51–56]. In addition, members of *Cohnella* have been reported as presenting the highest cellulolytic activity. For this reason, this genus is considered one of the most cellulolytic bacterial genera that have been isolated from compost and soil samples [57–59]. Concerning *Burkholderia* spp. isolated from forest soil or earthworm guts, Fujii et al. [60, 61] reported for the first time their cellulolytic and xylanolytic capacities. Several *Klebsiella* spp. are known to produce enzymes that break down cellulose and hemicellulose. For instance, *Klebsiella pneumoniae*, isolated from the gut of *Bombyx mori* (Lepidoptera: Bombycidae), was shown to possess cellulolytic and xylanolytic activities. [62]. Also, the cellulolytic bacterial strain *K*. *oxytoca* THLC0409 was used to produce ethanol from *P*. *purpureum* [63]. *Acinetobacter anitratus* was isolated from the haemolymph of an African snail and its endocellulase activity was quantitatively determined [64]. As we expected, most detected genera (*Cohnella*, *Klebsiella*, *Burkholderia*, *Paenibacillus*, *Acinetobacter* with the exception of *Roseomonas*) are known to have cellulase activity.

**Fig 7:**
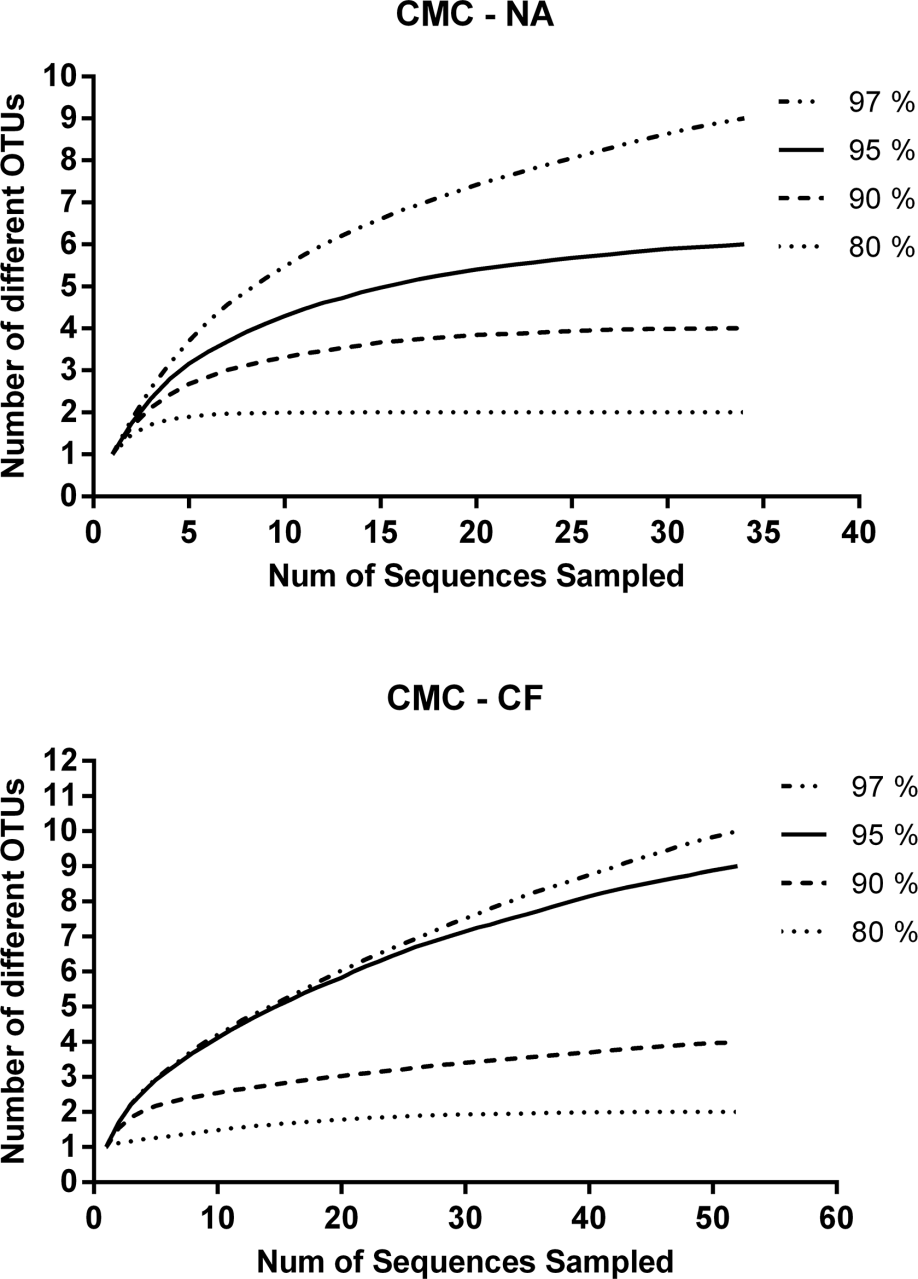
Rarefaction analysis of 16S rRNA gene sequences. (A) CMC—cultures from *N*. *aquilinus*. (B) CMC—cultures from *C*. *fulviceps*.

The rarefaction analysis indicated that the number of clones sequenced was sufficient to represent the bacterial diversity in the examined samples. A sequence similarity threshold of 80%, 90% and 95% has been considered to be differential at the phylum, class/family and genus level, respectively. A total of 35 sequences from CMC—cultures from *N*. *aquilinus* and 52 from CMC—cultures from *C*. *fulviceps* libraries were analyzed (Fig 7).

The CMC-CF clone library revealed a higher significant variability for both richness indices and for Simpson index in the case of measures of diversity (Table 2).

**Table 2:** Diversity and Richness indices calculated with the 16S rRNA gene sequence clone library^a^.

Sample	Sequences sampled	Number of OTUs	Richness indices		Diversity indices	
			Chao1	ACE	Shannon	Simpson
**CMC-NA**	34	9	10 (0.1–19.7)	11.7 (9.46–25.8)	1.9 (1.62–2.2)	0.16 (0.09–0.22)
**CMC-CF**	52	10	13.3 (10.5–32.1)	17.7 (11.4–52)	1.5 (1.2–1.8)	0.32 (0.21–0.43)

^a^ Diversity and richness indices were estimated based on 3% differences in nucleic acid sequence alignment. Values in parenthesis are 97% of confidence intervals were calculated by the Mothur program [34].

A comparison of the community composition across the two 16S rRNA gene sequence clone libraries using LIBSHUFF algorithm indicated significant differences (P<0.0001).

**Fig 8:**
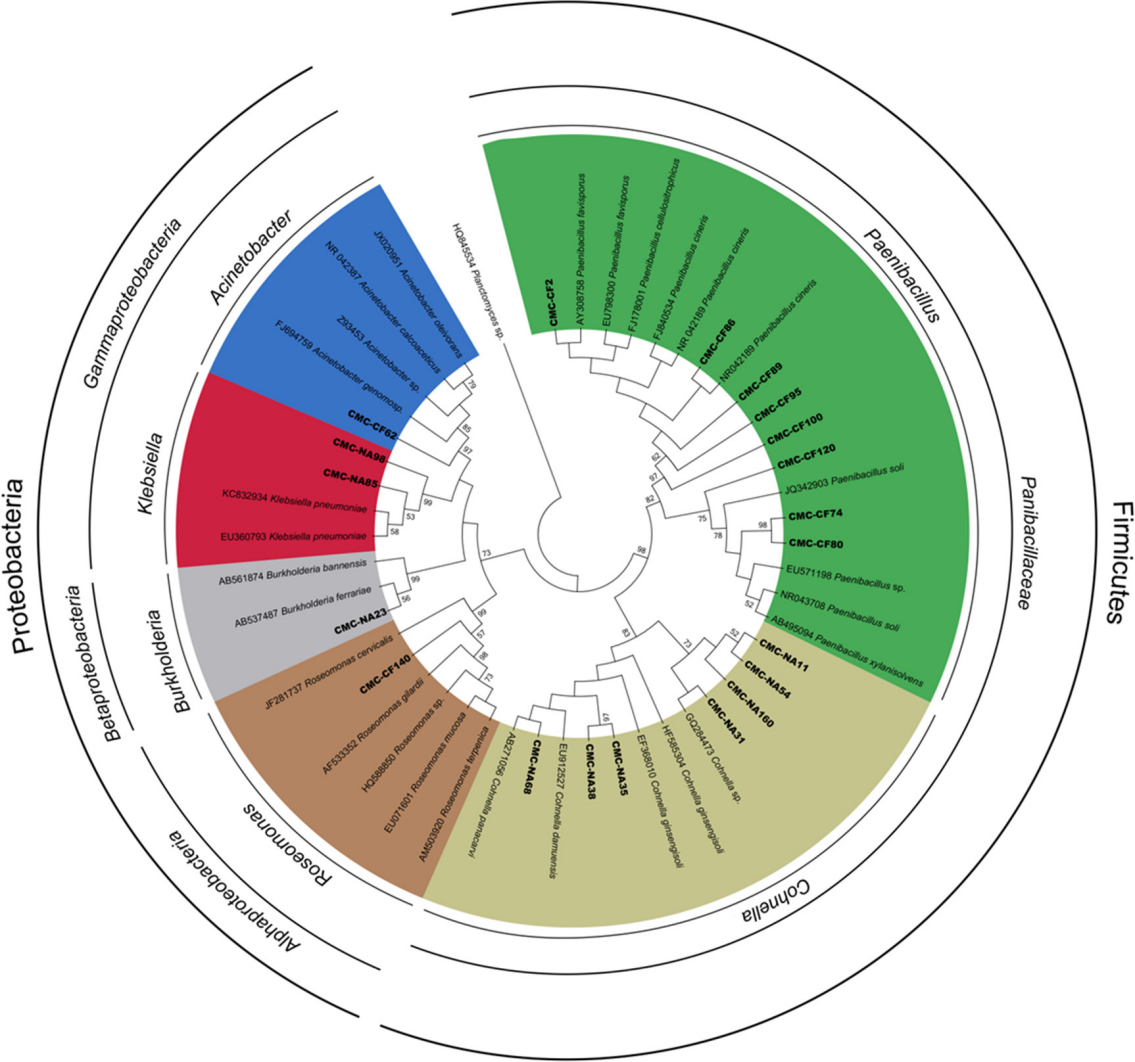
Phylogenetic tree generated from 16S rRNA gene sequences obtained in this study and representative sequences.

A phylogenetic analysis was performed to clarify the taxonomic position of 10 representative sequences obtained from cultures from *C*. *fulviceps* and 10 from cultures from *N*. *aquilinus* clone libraries. These sequences together with 29 additional reference sequences were used to construct a matrix and a dendrogram (Fig 8).

The phylogenetic analysis revealed two major clusters, corresponding to the phyla Firmicutes and Proteobacteria. Firmicutes were represented by two genera included in the family Paenibacillaceae: *Paenibacillus* and *Cohnella*. Members of *Paenibacillus* were present only in the CMC—cultures from *N*. *aquilinus* library, whereas those of the genus *Cohnella* were only in the CMC—cultures from *C*. *fulviceps* library. Regarding Proteobacteria, this cluster was subdivided into three groups: Alphaproteobacteria represented by the genus *Roseomonas*, Betaproteobacteria (genus *Burkholderia*), and Gammaproteobacteria (genera *Klebsiella* and *Acinetobacter*). Also in the case of Proteobacteria, each identified genus was associated to a single termite species (S1 Table). The distinct composition of the culturable gut microbiota of *N*. *aquilinus* and *C*. *fulviceps* could be attributed both to the phylogenetic distance between these termite genera and to their different diet preference. The intestinal bacterial community of termites is markedly conserved at the genus level, but differs significantly among host genera [65]. Gut microorganisms have also the ability to adapt themselves to changes in the insect diet, by induction of enzymes or by population changes in the microbial community [62, 66, 67].

A more comprehensive study involving metagenomic approaches which include non-culturable bacteria should be performed to elucidate this issue.

### Protein identification

The identification of the peptide sequences and the consortia member involved in the hydrolytic activities was performed by MALDI-TOF-TOF analysis.

The bands of the zymograms showing endoglucanase and/or xylanase activity (Fig 4) and a total of ten protein spots observed for SN–CMC–cultures from *N*. *aquilinus* on the 2D-gel (Fig 5) were excised for mass spectrometry sequencing. Seven of the ten protein spots could be identified by mass spectrometry. The identification of the proteins in each sample was performed by comparing the peptide sequence obtained from the comparative analysis against a Uniprot specific database of the genus present in the samples (*Cohnella*, *Klebsiella* and *Burkholderia*).

Table 3 displays the significant matches of each band or spot analyzed with the best protein accession, the percentage coverage of the peptides identified against the matching protein in each specific database. In addition, the best hits on the Blastp NCBI database were included in Table 3. When the UniProt database specific for *Cohnella* was used, several spots of SN–CMC–cultures from *N*. *aquilinus* (Fig 5) gave protein significant matches with xylanases and lipases. This result suggests that some of these proteins might be involved in cellulose hydrolysis and that this genus could be the most relevant regarding this activity (Table 3). However, when the *Klebsiella* and *Bulkholderia* specific databases were used, the best matches detected were for proteins with other functions, such as transcriptional regulation, secretion, cell division activation, or either they were uncharacterized. This could be because non-cellulolytic proteins are predominant in the analyzed gel bands or spots.

**Table 3:** Protein identification by mass spectrometry.

Gel spot/ Band number	Protein name/Description	Species	Protein accession	Theoretical MW (Da)	Peptide matches #	Peptide Matches*	Sequence Coverage (%)	Hit (NCBI Accesion number)	Score
**NA-CMC1**	Intracellular GH10 xylanase^a^	*Cohnella laeviribosi*	D5KTJ5_COHLA	39,477	4	R.LAGFAREHGMKMR.G	9	ADE08352.1	46.4
						R.HFNCITAENEMK.F			
						K.ALLYARLK.A			
						R.EHGMKMR.G			
	Uncharacterized protein^b^	*Klebsiella pneumoniae*	V3RIZ5_KLEPN	91,663	19	R.DYALSQIQEAFEAVDPRFFHLFEDEASVR.D	26	KDI84599.1	120
						R.AYGVTMMGSLGEVVSRAPDLR.S			
						R.YGGGALASMLGPVAGLVDDVVK.L			
	HlyD family secretion protein^c^	*Burkholderia thailandensis*	Q2T5R2_BURTA	51780	15	R.DEALLAAARNDVALAVAGVAAAQAKR.K	31	YP_439487.1	52.4
						R.IRLGEPADVRTDAYPSAVYR.G			
						R.KVIASLRPTSPRVPTMPNPR.R			
**NA-CMC3**	Lipase 3646^a^	*Cohnella* sp.	K7W8S6_9BACL	29, 660	3	R.GTSSTADWVSDALAYQIR.Y	16	AFW99795.1	67.2
						R.LEADNPGFCPVRRTGK.N			
						R.LLAAYTFGAPR.T			
	Transcriptional regulator^b^	*Klebsiella pneumoniae*	J2E8D0_KLEPN	9,871	10	R.MTAQGMQPK.S	63	WP_004147260.1	97.1
						R.RNAEAKLYSK.I			
						K.IIRMTAQGMQPKSIAR.I			
						R.MTAQGMQPKSIARIENCSVK.T			
	Uncharacterized protein^c^	*Burkholderia ambifaria*	B1T363_9BURK	17,145	8	K.VIASLRPTSPRVPTMPNPR.R	30	WP_006758180.1|	81.7
						R.KVIASLRPTSPRVPTMPNPR.R			
						R.IRLGEPADVRTDAYPSAVYR.G			
						R.DEALLAAARNDVALAVAGVAAAQAKR.K			
**NA-CMC4**	Intracellular GH10 xylanase^a^	*Cohnella laeviribosi*	D5KTJ5_COHLA	39,477	2	K.ALLYARLK.A	6	ADE08352.1	52.4
						R.LAGFAREHGMKMR.G			
	Uncharacterized protein^b^	*Klebsiella oxytoca*	H3MTZ9_KLEOX	21,771	6	R.MFAPTLSVAQSQQK.L	28	WP_004868981.1	67.7
						K.SMQVTWLPIQGPEQKAAKAK.A			
	Uncharacterized protein^c^	*Burkholderia* sp.	V5YPK5_9BURK	51,688	8	K.AGHPVTPVWLDNDRLK.L	19	WP_023842793.1	88.2
						K.EICGGMSGRLYMIGQGRLGR.V			
						R.VFNTYRPEVTMRDVLDNR.R			
						R.ETAALENWLDASHGKENSPR.H			
**NA-CMC5**	Pyridine nucleotide-disulfide oxidoreductase^b^	*Klebsiella pneumoniae*	W0YD74_KLEPN	16,032	2	R.GDAGEPAVPLRGCALRR.R	12	CDI25518.1	61.7
						K.RGDAGEPAVPLRGCALR.R			
	Putative uncharacterized protein^c^	*Burkholderia rhizoxinica*	E5AQI7_BURRH	6,783	2	R.SWRAVCVAGFCAVRR.G	25	WP_013435098.1	56.2
						R.RSWRAVCVAGFCAVR.R			
**NA-CMC7**	Intracellular GH10 xylanasea^a^	*Cohnella laeviribosi*	D5KTJ5_COHLA	39,477	1	R.LAGFAREHGMKMR.G	6	ADE08352.1	52.4
	Uncharacterized protein^b^	*Klebsiella pneumoniae*	W0XIF7_KLEPN	8,345	5	MVEVSLMLLNQTAMSK.V	57	WP_004192382.1	64.3
						R.VYHRHYCHTILSEGAIIR.A			
	Uncharacterized protein^c^	*Burkholderia* sp.	U2FAW4_9BURK	5044	3	MVRFVIVVVEEGIRR.V	81	WP_021163473.1	70.6
						R.RGTVGLLNAVGMSRDYSGWR.R			
						R.GTVGLLNAVGMSRDYSGWRR.R			
**NA-CMC8**	N-acetilglucosaminyltrasnferase^a^	*Paenibacillus* sp.	G3AD32_9BACL	22,529	1	R.GRPSDFGEDR.H	5	CCA94529.1	42.2
	Uncharacterized protein ^b^	*Klebsiella pneumoniae*	J2APE0_KLEPN	3,239	4	M.DQQVAHAIPR.A	100	WP_004152911.1	98.2
						MDQQVAHAIPRASK.S			
						K.STTTSPLVGNDWQLST			
	Uncharacterized protein^c^	*Burkholderia mallei*	Q62AV4_BURMA	25,352	8	R.CGGHPVAPPPDMVR.R	23	WP_020850392.1	60.0
						R.LAVFRSDDFDREPR.R			
						R.RCGGHPVAPPPDMVRR.R			
						R.CGGHPVAPPPDMVRRR.S			
**NA-CMC9**	MASE2 domain/diguanylate cyclase ^b^	*Klebsiella oxytoca*	K6JIX7_KLEOX	40,555	6	R.ELLEMQALMDPGLDLPNRR.F	11	WP_004136836.1	73.2
						R.RELLEMQALMDPGLDLPNR.R			
						K.NSIIEWIKEADEMLYQVK.R			
						R.RELLEMQALMDPGLDLPNRR.F			
						K.RRELLEMQALMDPGLDLPNR.R			
	Glyoxalase/bleomycin resistance protein/dioxygenase^c^	*Burkholderia cepacia*	J7JC15_BURCE	15,080	5	R.AMVRDPWGNTWQIATHRR.D	38	WP_014900262.1	55.4
						R.RAMVRDPWGNTWQIATHR.R			
						R.DAMPAFLYVYVENADSTYR.R			
**NA-PP-Xyl**	Lipase 3646^a^	*Cohnella* sp.	K7W8S6_9BACL	29, 660	1	R.GTSSTADWVSDALAYQIR.Y	6	AFW99795.1	67.2
	Cell division activator CedA ^b^	*Klebsiella pneumoniae*	Z5PJM2_KLEPN	12,647	7	R.IGGEGATNGATIGYDR.G.	64	AIK79843.1	86.3
						M.RIGGEGATNGATIGYDR.G			
						R.GFCFPLCLVNPFVMKPLR.Q			
						R.SPAFSVPESAQRWANQVRQEGEIEA			
	Enoyl-CoA hydratase^c^	*Burkholderia* sp.	I2DQA9_9BURK	26,952	9	M.AEIQVERADGVITITIARAAK.K	36	WP_014723615.1	80.5
						K.LLLGEPFDALEAHRIGIVNRV			
						K.ALLKDTGGVAVAARMAEEAAHFSAMLR.A			

^a^ *Cohnella* database (Uniprot)

^b^ *Klebsiella* database (Uniprot)

^C^ *Burkholderia* database (Uniprot)

*Only the longer peptide sequences matches were added

The absence of cellulolytic enzymes with high similarity to those described in the databanks could be further explained because of the methodology used for protein extraction [68]. According to Barnum et al. [69] some of the cellulolytic enzymes may be insoluble or may remain bound to the lignocelluloses matrix, which would make them difficult to be detected by this methodology.

Moreover, the identification of proteins is difficult because of the lack of genome sequence data to perform metaproteomics analysis. However, the result of the biodiversity analysis, described below, allowed the identification of peptides with similarity to the genus present in the sample of SN-CMC cultures from *N*. *aquilinus*.

Additional separation of these proteins by 2D-gel electrophoresis has allowed the isolation of protein spots of higher purity. This contributed to identify hemicellulolytic enzymes, as expected, since 2D gel is an optimal technique for high-resolution profiling of proteins with low abundance. Future studies will be focusing on their purification, characterization and overproduction as they could be of potential usage, together with other enzymes, to achieve the conversion of lignocellulose onto fermentable sugars.

### Molecular identification

The partial sequences of 16S rRNAs have been deposited in the GenBank database under accessions [GenBank: KJ933510- KJ933529].

## Conclusions

In this study, we have explored for the first time the (hemi) cellulolytic activities of enzymes present in two Argentinean native termites, *N*. *aquilinus* and *C*. *fulviceps*, and identified several peptides with similarity to xylanase by mass spectrometry sequencing. This paper provides new information on the cellulose degrading bacterial microbiota associated to these hosts. Endogenous and exogenous enzymes contained in total gut extracts and endosymbiont culture supernatants allowed the hydrolysis of *S*. *officinarum* bagasse and *P*. *purpureum*. Both biomasses are promising candidates as lignocellulosic feedsdtock for bioethanol production in South-America.

## Supporting Information

S1 Table List of 16S rRNA sequences obtained from CMC—cultures from *N*. *aquilinus* (CMC—NA) and CMC—cultures from *C*. *fulviceps* (CMC-FA) clone libraries. (DOC)
