# Supplementary material for: Prospection and Evaluation of (Hemi) Cellulolytic Enzymes Using Untreated and Pretreated Biomasses in Two Argentinean Native Termites
Source: PLoS One. 2015 Aug 27;10(8):e0136573. doi: 10.1371/journal.pone.0136573 (PMC4552170; doi:10.1371/journal.pone.0136573)
Supplement: S1 Table — (DOC) [file pone.0136573.s001.doc]

**S1 Table**. List of 16S rRNA sequences obtained from CMC - cultured from *N. aquilinus* (CMC - NA) and CMC - cultured from *C. fulviceps* (CMC-FA) clone libraries.

| **Clone number** | **Accession number** | **Closest relative**  **in database** | **Identity**  **(%)** | | **Genbank accession number** | |
| --- | --- | --- | --- | --- | --- | --- |
| CMC-NA 11  CMC-NA 23  CMC-NA 31  CMC-NA 35  CMC-NA 38  CMC-NA 54  CMC-NA 68  CMC-NA 85  CMC-NA 98  CMC-NA 160  CMC-CF 2  CMC-CF 62  CMC-CF 74  CMC-CF 80  CMC-CF 86  CMC-CF 89  CMC-CF 95  CMC-CF 100  CMC-CF 120  CMC-CF 140 | KJ933511  KJ933518  KJ933512  KJ933513  KJ933514  KJ933515  KJ933516  KJ933519  KJ933520  KJ933517  KJ933521  KJ933510  KJ933522  KJ933523  KJ933524  KJ933525  KJ933526  KJ933527  KJ933528  KJ933529 | *Cohnella* sp.  *Burkholderia ferrariae*  *Cohnella* sp.  *Cohnella damuensis*  *Cohnella* sp.  *Cohnella damuensis*  *Cohnella damuensis*  *Klebsiella pneumoniae*  *Klebsiella pneumoniae*  *Cohnella* sp.  *Paenibacillus favisporus*  *Acinetobacter genomosp*.  *Paenibacillus soli*  *Paenibacillus xylanisolvens*  *Paenibacillus cineris*  *Paenibacillus cineris*  *Paenibacillus cineris*  *Paenibacillus cineris*  *Paenibacillus favisporus*  *Roseomonas* sp. | | 97  97  97  96  96  96  95  99  99  98  98  99  96  96  99  97  99  99  95  96 | | GQ284473.1  AB537487.1  GQ284473.1  EU912527.1  GQ284473.1  EU912527.1  EU912527.1  KC832934.1  KC832934.1  GQ284473.1  EU798300.1  FJ694759.1  NR043708.1  AB495094.2  NR042189.1  NR042189.1  NR042189.1  NR042189.1  AY308758.1  HQ588850.1 |
